# Drug Therapy for Melanoma: Current Updates and Future Prospects

**DOI:** 10.3390/cancers18030382

**Published:** 2026-01-26

**Authors:** Hiroshi Kato

**Affiliations:** Department of Geriatric and Environmental Dermatology, Graduate School of Medical Sciences, Nagoya City University, 1-Kawasumi, Mizuho-cho, Mizuho-ku, Nagoya 467-8601, Japan; h-kato@med.nagoya-cu.ac.jp; Tel.: +81-52-853-8261

**Keywords:** melanoma, immune checkpoint inhibitors, targeted therapy, BRAF/MEK inhibition, treatment resistance

## Abstract

Melanoma was once considered ‘incurable’; however, drug therapy for the condition has dramatically transformed with the advent of immune checkpoint inhibitors and molecular targeted therapies. In this review, we summarize the published literature on melanoma drug therapy, presenting the current landscape of melanoma treatments, and discuss potential future transformations in melanoma therapy. Although the prognosis of advanced-stage melanoma had been extremely poor in the past, new-age immunotherapy has made long-term survival possible. Several immunotherapies and their combinations, as well as personalized vaccines, cell therapies, and intratumoral agents, have been tested with success; however, adverse toxicities have also been detected. Therefore, patient selection and management are critical. Furthermore, new approaches to overcome the limitations of the current treatments are also being developed. To implement these therapies clinically, guideline-recommended treatment algorithms should be followed while optimizing the therapies by considering factors such as presence of BRAF mutations which may lead to treatment resistance, increased disease burden/progression rate, toxicity tolerance, and the presence of brain metastases. In practice, the choice of the initial therapy should depend on the patient, leading to personalized therapy and minimal adverse effects.

## Simple Summary

Melanoma is an aggressive form of skin cancer that was previously challenging to treat once it spread to other parts of the body. Over the past decade, major advances in drug therapies have dramatically improved survival for many patients. In this review, we explain why new melanoma treatments are needed, we summarize the main types of modern therapies currently used in clinical practice, and we discuss promising new approaches under development. These include immune-based treatments which help the body recognize and attack cancer cells, targeted drugs that block specific genetic changes in tumors, and emerging strategies such as personalized vaccines and cell-based therapies. By outlining current treatment options, ongoing challenges such as drug resistance and side effects, and future research directions, we aim to help researchers and clinicians better understand how melanoma treatment is evolving and how these advances may continue to improve outcomes for patients.

## 1. Introduction

Melanoma is a highly malignant skin cancer with a poor prognosis when advanced. Prior to 2011, the 5-year survival rate for advanced melanoma was only approximately 6%, with a median overall survival of approximately 7–8 months [1]. However, over the past decade, the advent of immune checkpoint inhibitors (ICIs) and targeted kinase inhibitors has brought about a paradigm shift in treatment, dramatically improving patient survival [2]. For example, immunotherapy has extended median survival to approximately 5–6 years, and long-term follow-up from landmark trials has shown that the 10-year survival rate reaches approximately 50% with combination ICI therapy [3]. These advances have transformed metastatic melanoma from an almost invariably fatal disease to one in which sustained remission can be achieved in a significant proportion of patients. In this review, we summarize the published literature on melanoma drug therapy, assess the current landscape of melanoma treatments, and discuss potential future transformations in melanoma therapy.

## 2. ICIs in Melanoma

ICIs have revolutionized melanoma treatment by activating T-cell responses against tumors. This section outlines the latest evidence, including the mechanisms of action of ICIs, key clinical trial results, approved agents, and combination immunotherapy strategies.

### 2.1. Mechanism and Rationale for Checkpoint Inhibition

**Figure 1:**
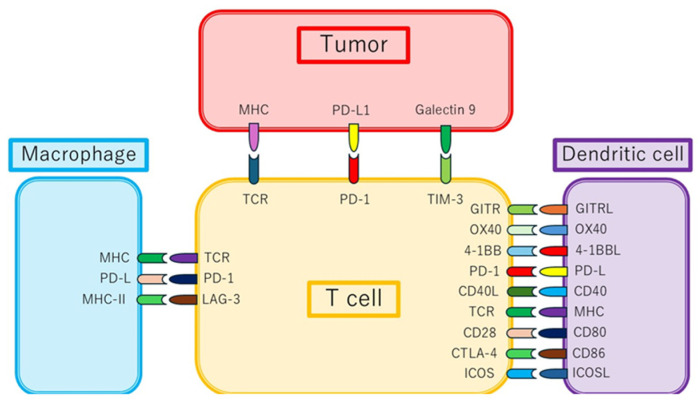
Immune checkpoint pathways in melanoma and melanoma-specific immune evasion contexts.

Under normal conditions, checkpoint molecules (CTLA-4, PD-1, and LAG-3) function as brakes in the immune system to maintain self-tolerance (Figure 1) [4]. Highly immunogenic melanomas exploit these checkpoints to evade immune attack [5]. ICIs that block CTLA-4 or PD-1 release these brakes and promote tumor destruction by T-cells. The strong rationale for checkpoint inhibition in melanoma is supported by its high mutational burden and frequent T-cell infiltration, which generate particularly high responsiveness to immunotherapy.

This schematic illustrates key interactions among tumor cells, T-cells, dendritic cells, and macrophages. It highlights the following:

- Inhibitory axes (e.g., PD-1/PD-L1, CTLA-4, LAG-3, and TIM-3);
- Co-stimulatory signals (e.g., CD28–CD80/86, OX40, 4-1BB, and GITR).

In melanoma, a high neoantigen load (often ultraviolet [UV]-driven in cutaneous melanoma) can promote T-cell recognition. However, tumors can evade immunity by upregulating PD-L1 through IFN-γ and by impairing antigen presentation, as well as by activating suppressive myeloid programs (TAMs/MDSCs) which limit the infiltration and function of effective T-cells. These melanoma-specific mechanisms offer a rationale for checkpoint blockade and rational combinations aimed at improving priming and trafficking, as well as overcoming resistance to the following checkpoint molecules:

PD-1 (Programmed Cell Death Protein 1); PD-L1 (Programmed Cell Death Ligand 1); CTLA-4 (Cytotoxic T-Lymphocyte Antigen 4); LAG-3 (Lymphocyte Activation Gene-3); TIM-3 (T-cell Immunoglobulin and Mucin-domain-containing-3); CD28 (Cluster of Differentiation 28); CD80/86 (Cluster of Differentiation 80/86); OX40 (Tumor Necrosis Factor Receptor Superfamily Member 4); 4-1BB (TNF Receptor Superfamily Member 9); GITR (Glucocorticoid-Induced TNFR-Related Protein); IFN-γ (Interferon Gamma); TAMs (Tumor-Associated Macrophages); MDSCs (Myeloid-Derived Suppressor Cells).

### 2.2. Clinical Efficacy of Checkpoint Inhibitors: Key Trials and Outcomes

#### 2.2.1. Anti-CTLA-4 (Ipilimumab)

Ipilimumab was the first ICI to demonstrate improved survival in patients with melanoma, showing a survival benefit over peptide vaccines and chemotherapy in trials conducted between 2010 and 2011 [6]. It showed durable responses in some patients (objective response rate of approximately 10–15%) and led to long-term remission in a small number of cases. However, many patients did not respond, and immune-related adverse events (irAEs) were significant, prompting the search for safer and more effective checkpoint inhibitors.

#### 2.2.2. Anti-PD-1 (Pembrolizumab, Nivolumab)

PD-1 inhibitors have demonstrated higher efficacy than ipilimumab in advanced melanoma. In the Phase III KEYNOTE-006 trial, pembrolizumab doubled the response rate compared to ipilimumab and significantly improved overall survival. At the 5-year follow-up, the survival rate was approximately 40% in the pembrolizumab group and 31% in the ipilimumab group [7]. Similarly, nivolumab outperformed ipilimumab in the CheckMate-066/067 trials [8,9]. PD-1 monotherapy achieves response rates of approximately 30–45% and exhibits a more favorable toxicity profile than CTLA-4 inhibitors [10]. Long-term data have also shown favorable outcomes. For example, a recent follow-up study reported that approximately 34% of patients treated with pembrolizumab survived for 10 years after treatment [7].

#### 2.2.3. Combination of Checkpoint Inhibitors

The combination of ipilimumab and nivolumab has demonstrated synergistic effects. In the CheckMate-067 trial, nivolumab plus ipilimumab achieved a higher response rate (approximately 58%) and longer progression-free survival than monotherapy, although toxicity increased [9]. Notably, updated 6.5-year and 10-year results reported a median overall survival of approximately 6 years for combination therapy, the longest ever reported for melanoma treatment [11]. Approximately 48–50% of patients in the combination therapy group survived for 5 years, and approximately half achieved disease control for 10 years. This sustained efficacy established the combination of CTLA-4 and PD-1 inhibitors as a standard first-line treatment for eligible patients. In 2022, the combination of nivolumab and relatlimab was approved by the United States Food and Drug Administration (FDA). The RELATIVITY-047 trial demonstrated improved progression-free survival (PFS) (median PFS 10.1 months vs. 4.6 months) with the addition of relatlimab to nivolumab [12]. This suggests that PD-1/LAG-3 combination therapy, by targeting an alternative T-cell exhaustion pathway, may induce responses even in patients resistant to PD-1 monotherapy.

### 2.3. Resistance to ICIs

Although anti-PD-1 and anti-CTLA-4 therapies have led to durable responses in some patients, primary and acquired resistance remain major barriers in advanced melanoma. Notably, resistance is rarely driven by a single mechanism. Rather, it reflects a dynamic interplay between tumor-intrinsic alterations and an evolving tumor microenvironment (TME). Understanding these mechanisms helps explain why many rational combinations yield heterogeneous benefits and guides trial design toward biomarker-enriched or mechanism-based strategies.

#### 2.3.1. Tumor-Intrinsic Mechanisms

Impaired antigen presentation is a canonical tumor-intrinsic driver of ICI resistance. The loss or downregulation of components involved in antigen processing and presentation, such as HLA class I molecules or β2-microglobulin (B2M), can render tumor cells less visible to cytotoxic T lymphocytes. This limits the efficacy of T-cell-dependent immunotherapies. In parallel, defects in interferon-γ (IFN-γ) signaling can blunt immune-mediated tumor control. Alterations affecting the responsiveness of the IFN-γ pathway may reduce antigen presentation, decrease the production of chemokines required for T-cell recruitment, and weaken the amplification loop which sustains inflamed (“hot”) tumor states.

Additionally, oncogenic signaling pathways can shape immune escape. MAPK pathway reactivation and PI3K–AKT signaling, which are well-recognized in targeted therapy resistance, may also promote immunosuppression through multiple routes. These include reduced T-cell infiltration, an altered cytokine/chemokine milieu, and metabolic competition within the TME. These tumor-intrinsic programs can contribute to a non-inflamed phenotype and can help explain why biologically plausible combinations do not consistently result in meaningful clinical benefit without appropriate patient selection and optimized scheduling.

#### 2.3.2. Microenvironmental Mechanisms

Even when tumor cells remain antigenic, the surrounding microenvironment may prevent effective anti-tumor immunity. One major pattern is T-cell exclusion or dysfunctional infiltration, in which T-cells are either absent from the tumor core or present but ineffective. Immunosuppressive myeloid populations, particularly myeloid-derived suppressor cells (MDSCs) and tumor-associated macrophages (TAMs), can inhibit T-cell function through checkpoint ligands, arginine depletion, reactive oxygen species, and immunosuppressive cytokines.

Cytokine networks and soluble mediators further reinforce resistance. Elevated immunosuppressive factors (e.g., TGF-β, IL-10, and other inhibitory signals) can limit effector T-cell activity and promote regulatory programs. Together, these mechanisms create a TME in which checkpoint blockade alone is insufficient. This supports the rationale for approaches which (i) promote T-cell priming and trafficking, (ii) remodel suppressive myeloid states, or (iii) deliver effective cytotoxicity independent of endogenous T-cell recognition.

#### 2.3.3. Clinical Implications and Therapeutic Positioning

From a clinical perspective, separating resistance into primary and acquired forms is beneficial because the therapeutic logic can differ. Primary resistance often reflects a non-inflamed TME or tumor-intrinsic immune invisibility, favoring strategies that initiate or broaden anti-tumor immunity (e.g., rational combinations, intratumoral agents that convert “cold” to “hot,” vaccines, or cellular therapies). In contrast, acquired resistance after a period of response may involve immune editing, adaptive pathway rewiring, or antigen-presentation defects. This motivates strategies such as switching immunotherapy classes, incorporating local control for oligoprogression, or enrolling in trials targeting specific escape mechanisms.

These considerations also help in interpreting the mixed results from combination trials. Heterogeneous enrollment (e.g., baseline T-cell-inflamed versus non-inflamed states, tumor burden, lactate dehydrogenase (LDH) elevation, and brain metastases) and nonbiomarker-driven designs can dilute the true benefits in responsive subgroups while increasing toxicity. Therefore, future studies should prioritize mechanism-based endpoints, biomarker-enriched cohorts, and subtype-aware stratification. The goal is to increase response rates and to sustain durable disease control with acceptable feasibility in routine practice.

### 2.4. Use as Adjuvant and Neoadjuvant Therapy

ICIs have advanced beyond the treatment of advanced disease to include use as adjuvant therapy for melanoma. Anti-PD-1 therapy (nivolumab or pembrolizumab) is the standard adjuvant treatment for resected stage II/III/IV melanoma and significantly improves recurrence-free survival [13,14]. Combination therapy with ipilimumab and nivolumab has demonstrated efficacy as a neoadjuvant therapy in clinical trials (pathological response rate of approximately 50–60%) [15]. Multiple trials aimed at optimizing neoadjuvant immunotherapy are ongoing; however, these are not yet considered standard treatments.

## 3. Molecular Targeted Therapy for Melanoma

Targeted therapy has become a cornerstone of treatment, particularly for patients with melanoma harboring treatable mutations, such as BRAF gene mutations. This section discusses the latest evidence regarding molecular targeted agents such as BRAF and MEK inhibitors, issues of resistance, and combination strategies to prolong their efficacy.

### 3.1. BRAF and MEK Inhibition

#### 3.1.1. Mechanism and Clinical Evidence

Approximately 20–40% of cutaneous melanomas (varying by race and site of origin; expression is rare in acral melanomas) harbor activating mutations in BRAF (the most frequent being V600E/K). These mutations lead to the constitutive activation of the MAPK/ERK pathway and promote tumor growth [16]. This discovery led to the development of selective BRAF and MEK inhibitors that block this pathway. BRAF inhibitors (vemurafenib, dabrafenib, and encorafenib) have demonstrated remarkable initial efficacy in BRAF-mutated melanoma, with high response rates and improved overall survival [17,18,19]. However, responses to single-agent BRAF inhibitors are often transient, with a median PFS of approximately 6–7 months, owing to the rapid development of resistance [20].

To enhance durability, dual inhibition of BRAF and MEK was introduced. The addition of an MEK inhibitor (trametinib, cobimetinib, or binimetinib) to a BRAF inhibitor provides a more comprehensive blockade of the MAPK pathway and delays the emergence of resistance. Landmark clinical trials (COMBI-D, COMBI-V, coBRIM, and COLUMBUS) demonstrated that combination therapies (dabrafenib plus trametinib, vemurafenib plus cobimetinib, and encorafenib plus binimetinib) improved response rates and prolonged survival compared with BRAF-inhibitor monotherapy [21,22,23]. For example, a combination of dabrafenib and trametinib achieved a 5-year overall survival rate of approximately 34% in previously untreated patients who were BRAF-mutation-positive (compared with approximately 22% with BRAF-inhibitor monotherapy) [21]. These combination therapies have become the standard treatment for BRAF-mutant metastatic melanoma, inducing rapid tumor shrinkage in the majority of patients and significantly improving median survival. Targeted therapy also plays a role in the adjuvant setting. For example, the COMBI-AD trial demonstrated that administering dabrafenib plus trametinib for 12 months as adjuvant therapy in patients with resected stage III BRAF-mutant melanoma significantly improved recurrence-free survival and overall survival compared with a placebo, leading to its approval for adjuvant use [24].

Despite their efficacy, acquired resistance to BRAF/MEK inhibitors remains universal. Melanoma cells develop evasion mechanisms such as reactivation of the MAPK/ERK pathway (via secondary NRAS or MEK mutations, BRAF splice variants, etc.) or activation of alternative survival pathways (e.g., PI3K–AKT) [25]. These mechanisms allow tumor growth to resume despite ongoing BRAF/MEK inhibition. Clinically, most patients receiving BRAF/MEK therapy experience tumor progression within approximately 9–15 months. Several strategies have been explored to address these issues.

#### 3.1.2. Next-Generation Inhibitors

Novel ERK and pan-RAF inhibitors aim to overcome resistance by targeting downstream nodes or allosteric mechanisms [26]. Early trials of ERK inhibitors (e.g., ulixertinib) showed some activity against BRAF-inhibitor-resistant melanoma; however, toxicity and optimal dosing remain challenging.

#### 3.1.3. Combination Therapy with Additional Pathway Inhibitors

Triple combination therapies that add a third agent to BRAF and MEK inhibition to block bypass pathways are currently under investigation. Examples include adding a PI3K or AKT inhibitor to BRAF/MEK inhibition [27] or adding a CDK4/6 inhibitor based on the role of the CDK4 pathway in melanoma cell cycle progression [28]. Although some combination therapies are limited by toxicity, research continues to identify tolerable regimens capable of extending disease control duration.

#### 3.1.4. Intermittent Dosing Strategies

Preclinical models suggest that intermittent administration of BRAF inhibitors may delay the development of resistance by avoiding continuous pathway-reactivation pressure. Pilot studies have evaluated intermittent dosing schedules for dabrafenib and trametinib, showing the potential to extend response duration in specific cases, although this is not currently the standard therapy [29].

#### 3.1.5. Combination of Targeted Therapy and Immunotherapy

This is a novel approach combining the rapid tumor shrinkage effect of targeted therapy with sustained immune memory induced by immunotherapy. Early-phase trials of concurrent BRAF/MEK inhibitors and anti-PD-1 antibodies have demonstrated high response rates. For example, the IMspire150 trial showed improved progression-free survival with atezolizumab (a PD-L1 antibody) plus vemurafenib and cobimetinib compared with targeted therapy alone [30]. However, other studies (the COMBI-i trial using spartalizumab and the KEYNOTE-022 trial using pembrolizumab) have yielded conflicting results regarding whether the triple combination therapy confers superior long-term survival and has the potential for increased toxicity [31,32]. Why have triplet strategies produced inconsistent benefits? Differences in patient risk profiles (tumor burden, LDH levels, and brain metastases), prior exposure, and endpoints across studies may partly explain the heterogeneity in outcomes. Furthermore, overlapping toxicities often lead to dose interruptions, which may blunt sustained immune priming. These findings suggest that triplet strategies should be reserved for carefully selected patients in clinical trials or for specific high-risk scenarios. Biomarker-driven enrichment and optimized scheduling (e.g., induction/lead-in approaches) are key next steps.

### 3.2. Other Molecular Targets and Therapies

Although BRAF is the primary target in melanoma, other molecular subtypes have also been studied (Table 1).

#### 3.2.1. NRAS-Mutated Melanoma

NRAS mutations (found in approximately 15–20% of melanomas) activate the MAPK pathway, but no direct NRAS inhibitors exist.

#### 3.2.2. MEK Inhibitors

MEK inhibitors (e.g., binimetinib) show some activity against NRAS-mutated melanoma, with phase III trials demonstrating improved progression-free survival compared with chemotherapy; however, no overall survival benefit was achieved [33]. Combination strategies (e.g., MEK and CDK4/6 inhibitors) are being explored for NRAS-mutated tumors. Targeted therapy for NRAS-mutated melanoma remains an unmet need, and these patients often rely on immunotherapy as first-line treatment.

#### 3.2.3. c-KIT Mutations

Activating mutations or amplifications of KIT are found in acral and mucosal melanomas. In these relatively rare cases, c-KIT inhibitors, such as imatinib, may induce a therapeutic response. Small trials reported imatinib response rates in KIT-mutated melanoma of 38% for the mucosal type, 25% for the acral lentiginous type, and 8% for melanoma of unknown primary [34]. Newer KIT inhibitors (nilotinib and dasatinib) have also been used off-label with limited success [35]. Because of the rarity of KIT mutations, these therapies are generally considered for refractory cases or in clinical trials.

#### 3.2.4. NTRK Fusion Genes and Other Rare Targets

NTRK gene fusions are rarely found in melanomas (particularly in Spitz-like melanomas) [36]. TRK inhibitors (larotrectinib and entrectinib) have been approved for all TRK-fusion-positive solid tumors and yield dramatic responses in this molecularly defined subset. Similarly, rare non-V600 BRAF mutations (e.g., BRAF fusions or exon 11/15 mutations) may not respond to standard BRAF inhibitors, but could be targets in clinical trials of MEK inhibitors or next-generation RAF inhibitors; case reports occasionally describe responses [37].

#### 3.2.5. Angiogenesis and Other Pathways

Anti-angiogenic agents (e.g., VEGF pathway inhibitors) generally show limited efficacy in melanoma; however, combination therapies are being explored to modulate the TME. For example, trials combining bevacizumab (an anti-VEGF agent) with checkpoint inhibitors have been conducted but have not achieved definitive success [38]. Efforts targeting other pathways (MET, PTEN/PI3K, etc.) are primarily ongoing in clinical trials of melanoma subgroups harboring these abnormalities [39].

## 4. Novel and Investigational Therapies

New breakthrough therapies for melanoma are currently under development. This section covers novel and investigational treatments, including next-generation immunotherapies, small-molecule compounds, bispecific antibodies, oncolytic viruses, and other approaches under development. These emerging strategies aim to further improve treatment outcomes, particularly in patients who have exhausted current treatment options or have resistant diseases. Key translational barriers include (i) toxicity and feasibility in routine practice, (ii) variability of efficacy across trials and populations, and (iii) limited generalizability owing to eligibility constraints (e.g., HLA restriction, injectable lesions, specialized manufacturing). Therefore, these approaches should be interpreted as promising but not yet broadly implementable, pending phase III validation and optimization of patient selection.

### 4.1. New Immune Checkpoint Targets

Following the success of PD-1 and CTLA-4 inhibitors, additional checkpoint receptors have been targeted. Agents against TIGIT, TIM-3, and other proteins are currently in advanced-stage clinical trials, often in combination with PD-1 inhibitors [40,41]. For example, anti-TIGIT antibodies are being tested in combination therapy for melanoma to determine whether they can enhance responses [41]. Early signs in other cancer types suggest their potential, but their role in melanoma requires further evaluation. These next-generation checkpoint inhibitors may address additional aspects of T-cell exhaustion and benefit patients who do not fully respond to PD-1/CTLA-4 inhibition.

### 4.2. Cytokine Therapy (IL-2/IL-21 and Beyond)

High-dose IL-2 is an older immunotherapy for melanoma that occasionally produces durable remission but is associated with severe toxicity. New formulations aim to harness cytokine stimulation while reducing toxicity. NKTR-214 (benpegaldesleukin) is a pegylated IL-2 variant that advanced to Phase III trials in combination with nivolumab but failed to meet its primary endpoint [42]. Trials are ongoing with other modified cytokines (IL-2, IL-21, and IL-15 superagonists) and STING agonists administered intratumorally to stimulate local immune attack [43].

### 4.3. Personalized Cancer Vaccines

Therapeutic cancer vaccines have historically achieved limited success in melanoma; however, this field has been revitalized using messenger ribonucleic acid (mRNA) vaccine technology. A recent breakthrough is the combination therapy of a personalized neoantigen mRNA vaccine, mRNA-4157 (V940), with pembrolizumab. In a Phase IIb trial (KEYNOTE-942) targeting high-risk resected melanoma, adding the mRNA vaccine to pembrolizumab improved recurrence-free survival, reducing the risk of recurrence or death by approximately 44–50% compared with pembrolizumab alone [44]. At 18 months, the disease-free survival rates were 79% and 62% in the vaccine combination and pembrolizumab monotherapy groups, respectively. This personalized vaccine, designed based on tumor mutations in each patient, is currently undergoing Phase III trials. If these results are sustained, this approach could become the first cancer vaccine approved for melanoma. Similarly, other vaccine approaches, including peptide and deoxyribonucleic acid (DNA) vaccines, are also being tested, often aimed at enhancing the efficacy of checkpoint inhibitors.

### 4.4. Adoptive Cell Therapy (Tumor-Infiltrating Lymphocytes [TILs] and T-Cell Receptors [TCRs])

Although not a “drug” in the conventional sense, cell therapy is an emerging treatment worthy of attention. TIL therapy involves expansion and reinfusion of tumor-infiltrating T-cells and has demonstrated durable responses in refractory melanoma (approximately a 36% overall response rate in Phase II trials, with some complete responses observed) [45]. If approved, TIL therapy could become an option following failure of immunotherapy and targeted therapy. Furthermore, modified TCR therapies targeting melanoma antigens (such as gp100) are under investigation, and a TCR-based therapy targeting MAGE-A4 (afamitresgene autoleucel [afami-cel]) is under development [46]. Personalized cell therapies represent a new frontier that complements drug-based approaches.

### 4.5. Bispecific Antibodies and T-Cell Engagers

Bispecific T-cell engagers represent an innovative class of immunotherapies that physically link T-cells to tumor cells. Tebentafusp is a prime example of a bispecific molecule (half TCR, which targets the gp100 melanoma antigen, and half anti-CD3, which captures T-cells) that has demonstrated improved survival in metastatic uveal melanoma [47]. In a Phase III trial of patients with human leukocyte antigen (HLA)-A*02:01-positive uveal melanoma, tebentafusp significantly improved overall survival compared with standard therapy (median overall survival, 21.7 months vs. 16.9 months; hazard ratio, 0.68). The 3-year survival rate was 27% in the tebentafusp group and 18% in the control group. This is the first treatment to extend survival in patients with uveal melanoma, which is often resistant to conventional immunotherapy. Other bispecific antibodies (sometimes called ImmTACs or BiTEs) targeting antigens such as MELANA/MART-1 and PRAME have also been investigated in melanoma [48]. The promise of these agents lies in their ability to directly mobilize and activate T-cells at the tumor site, even when the patient’s endogenous T-cell response is weak. This strategy may be particularly beneficial in patients who do not respond to checkpoint inhibitors.

### 4.6. Oncolytic Viruses and Intratumoral Therapy

Melanoma was the first solid tumor for which oncolytic viral therapy was approved. Talimogene laherparepvec (T-VEC), a genetically modified herpes simplex virus that selectively infects and kills tumor cells while expressing GM-CSF, has been approved for the treatment of injectable, unresectable melanoma (typically locally advanced stage III lesions) [49]. T-VEC induces tumor regression at the injection site by stimulating systemic immunity and may shrink lesions at non-injection sites. As a monotherapy, T-VEC did not prolong overall survival in metastatic melanoma but improved durable-response rates compared with GM-CSF injections. Recent studies have focused on combining oncolytic viruses with immunotherapy. For example, a randomized Phase II trial showed that the T-VEC plus ipilimumab combination group had a higher objective response rate (approximately 39% vs. 18%) than the ipilimumab monotherapy group, with no additional toxicity observed [50]. The response in the combination group was more durable, persisting beyond 5 years. Similarly, a trial combining T-VEC with pembrolizumab showed improved response rates compared with pembrolizumab alone, suggesting that tumor-lytic virus injection may enhance checkpoint inhibition by immunologically “activating” (‘warming’) “cold” tumors [51]. Beyond T-VEC, numerous oncolytic viruses are under development (e.g., Coxsackievirus A21 and vaccinia virus-based vectors) [52,53]. Intratumoral immunotherapies, such as TLR9 agonists and IL-12 cytokine vectors, are also being tested for melanoma. These intralesional therapies may be particularly useful for patients with accessible tumors (e.g., intermediate metastases) and could help induce systemic antitumor immunity while limiting systemic side effects [54].

### 4.7. PARP Inhibitors

Interest exists in PARP inhibitors for certain subsets of melanoma with homologous recombination deficiency or for use as radiosensitizers in melanoma brain metastases. Recent exploratory studies suggest that some patients with melanoma may benefit from PARP inhibitors after failure of BRAF/MEK therapy, although this approach is not yet standard [55].

### 4.8. Metabolic and Epigenetic Targets

Melanoma cells rewire their metabolic pathways to survive treatment. Drugs targeting metabolic checkpoints (e.g., IDO pathway inhibitors) and epigenetic modifiers (e.g., HDAC inhibitors) have been explored. No clear clinical success has been achieved in this area [56,57]. The lack of definitive efficacy likely stems from pathway redundancy, insufficient biomarker enrichment, and dose-limiting toxicities which hinder sustained target inhibition. Future trials should be conducted to identify molecular or immune context biomarkers (e.g., pathway activation signatures) and to focus on rational combinations with clear mechanistic endpoints rather than on unselected populations.

### 4.9. Microsatellite Instability-High (MSI-H) Melanoma

A very small proportion of melanomas are MSI-H. These tumors respond well to PD-1 inhibitors (similarly to other MSI-H cancers), and research is ongoing to determine whether combination therapies, such as PD-1 and CTLA-4 inhibitors, could further improve cure rates in this subgroup [58].

### 4.10. PAI-1 Inhibitors

Furthermore, the TM5614-MM trial conducted in Japan evaluated the combination of the PAI-1 inhibitor TM5614 and nivolumab in patients with advanced melanoma resistant to anti-PD-1 antibodies. The response rate was 29.4%, indicating efficacy even in anti-PD-1 nonresponders (*p* = 0.027). These results suggest new possibilities for immune reactivation strategies [59].

## 5. Melanoma Subtypes and Implications for Systemic Therapy

Melanoma is a biologically heterogeneous group of diseases, not a single entity. Much of the pivotal evidence that informed modern systemic therapy was generated from studies in cutaneous melanoma; however, acral, mucosal, and uveal melanomas have distinct etiologies, genomic landscapes, immune microenvironments, and clinical behaviors. These differences are clinically relevant because they influence

(i) The expected magnitude of benefit from ICI;
(ii) The prevalence of actionable driver alterations, thus influencing the feasibility of targeted therapy;
(iii) How biomarkers such as tumor mutational burden (TMB), PD-L1 expression, and circulating tumor DNA (ctDNA) should be interpreted. Therefore, evidence derived from cutaneous melanoma should not be uncritically generalized to non-cutaneous subtypes. Therefore, subtype context must be explicitly considered in treatment selection and trial design.

### 5.1. Cutaneous Melanoma: Ultraviolet-Driven Genomics and Higher Likelihood of ICI Benefit

Cutaneous melanoma is commonly characterized by ultraviolet (UV)-associated mutagenesis, which contributes to a higher mutational burden and increased neoantigen formation. This biological background provides a plausible mechanistic basis for the favorable activity of ICIs generally observed in landmark trials. From a precision oncology perspective, cutaneous melanoma contains a higher frequency of MAPK pathway alterations, including BRAF V600 mutations and other driver events (e.g., NRAS and NF1). This has enabled well-established targeted strategies, such as BRAF/MEK inhibition, in molecularly defined subsets. In practice, the setting of the subtype in which ICIs and BRAF/MEK inhibitors have robust phase III evidence creates a clinically meaningful “sequence problem,” in which disease tempo, tumor burden, brain metastasis status, LDH elevation, and patient comorbidities are often used to individualize upfront therapy. Notably, even within cutaneous melanoma, heterogeneity persists (e.g., varying degrees of T-cell infiltration and immune evasion), which partly explains why biomarkers such as PD-L1 and TMB show only modest predictive performance at the level of the individual patient.

### 5.2. Acral and Mucosal Melanoma: Distinct Drivers, Lower TMB, and Different ICI Expectations

Acral and mucosal melanomas are biologically distinct from UV-driven cutaneous melanoma. They tend to exhibit lower TMB on average, and they are enriched for structural variations and alternative oncogenic drivers. A comparatively higher prevalence of KIT alterations is observed in subsets. Clinically, these features often translate into different patterns of responses to systemic therapy. Although ICIs remain a critical option, the magnitude and consistency of the benefits reported in cutaneous melanoma may not be fully recapitulated in acral or mucosal disease. This gap has several implications.

First, patient counseling should acknowledge the uncertainty and variability in ICI outcomes across subtypes. Clinicians should also consider early radiologic reassessment and timely treatment adaptation for aggressive disease courses. Second, actionable BRAF V600 mutations are less frequent in acral and mucosal melanoma. Consequently, access to “standard” BRAF/MEK targeted therapy is limited at the population level. Broad genomic profiling is valuable for identifying less common but actionable alterations (e.g., KIT) and for facilitating clinical trial enrollment. Third, biomarker interpretation requires nuance. A “low” TMB in acral or mucosal melanoma may be a subtype-associated baseline rather than an individual marker of immune resistance. PD-L1 expression, although biologically informative, has not been established as a decisive test for selecting ICIs in routine practice. Overall, a subtype-aware approach to trial design is essential to generate clearer evidence in these populations. This approach includes stratification by subtype, biomarker-enriched cohorts, and endpoints tailored to expected response kinetics.

### 5.3. Uveal Melanoma Has Distinct Oncogenic Biology and Subtype-Specific Therapeutic Paradigms

Uveal melanoma is the most distinct subtype in terms of oncogenic drivers, metastatic patterns, and immune contexture. Its molecular pathogenesis is commonly linked to alterations that differ fundamentally from those dominant in cutaneous melanoma, and its TME often displays features that may limit responsiveness to conventional ICIs. Clinically, this has historically translated to less benefit and consistency with standard ICI regimens than with cutaneous melanoma, underscoring the need for subtype-specific approaches.

A key example is tebentafusp, a T-cell-engaging bispecific therapy directed against gp100 in the context of HLA-A*02:01. The requirement for a specific HLA type demonstrates that “clinically actionable biomarkers” in melanoma can extend beyond tumor genomics and can directly determine access to effective systemic therapy on a subtype-specific basis. This paradigm highlights two broader lessons: First, uveal melanoma should be considered separately from cutaneous melanoma based on dedicated evidence rather than extrapolation. Second, eligibility constraints (e.g., HLA restriction, specialized manufacturing, or limited-access platforms) should be transparently presented to indicate real-world feasibility. Moving forward, expanding T-cell-redirecting strategies and antigen-directed approaches may provide opportunities to expand subtype-specific treatment options. However, their clinical value depends on confirmatory trials, toxicity management, and scalable implementation.

### 5.4. Practical Implications: Avoiding Overgeneralization and Aligning Biomarkers with Clinical Decisions

Taken together, these subtype differences have immediate practical consequences. In clinical writing and decision-making, distinguishing between the following is beneficial: (a) treatments supported by high-level evidence primarily in cutaneous melanoma (where generalizability to non-cutaneous subtypes is uncertain); (b) subtype-enriched targeted opportunities (e.g., KIT-directed therapy in selected acral/mucosal cases); and (c) subtype-specific standards (e.g., tebentafusp in eligible uveal melanoma). Likewise, biomarkers should be interpreted through the lens of subtype biology. TMB and PD-L1 are better viewed as context-dependent risk/response modifiers than as gatekeeping tests. However, certain biomarkers (e.g., BRAF V600 status and HLA-A*02:01 for tebentafusp eligibility) directly determine treatment availability. Throughout this review, we explicitly indicate when evidence is mainly derived from cutaneous melanoma and we highlight areas where data on acral, mucosal, or uveal melanoma are limited, conflicting, or emerging.

## 6. Latest Trends in Clinical Guidelines

Modern clinical guidelines (NCCN, ESMO, ASCO) are evolving to reflect these new therapies and evidence. Although the recommendations of the NCCN, ESMO, and ASCO are largely concordant in the core pillars of systemic therapy—namely, anti-PD-1-based immunotherapy as a backbone, BRAF/MEK inhibition for BRAF V600-mutant disease, and the use of adjuvant anti-PD-1 therapy (and adjuvant BRAF/MEK therapy in selected BRAF-mutant cases)—they differ in how the guidance is framed and in the degree of granularity for specific scenarios. NCCN typically presents multiple acceptable options and grades them using category designations, which can be practical for real-world decision-making when several regimens are reasonable. ESMO emphasizes evidence synthesis and clinical nuance. This includes a clearer discussion of patient selection criteria, such as disease tempo, tumor burden, LDH elevation, and CNS involvement, as well as feasibility considerations. This approach may result in more conservative language for strategies with evolving evidence. The ASCO guidelines, although substantially overlapping in recommended regimens, frequently focus on evidence quality and strength of recommendations, and they may adopt more standardized phrasing aimed at general oncology practice. The areas where the three guidelines differ most in tone include the level of endorsement for emerging combinations (often positioned as investigational or for selective use), how sequencing is handled when immunotherapy and targeted therapy are options, and the degree of detail provided for special populations (e.g., brain metastases and rare subtypes). Therefore, rather than treating these guidelines as conflicting, clinicians can interpret them as complementary. NCCN can be used for option sets and practical pathways, ESMO for contextual nuance and feasibility, and ASCO for evidence-graded recommendations, while acknowledging that updates may occur as pivotal trial data mature. The current consensus in global guidelines, highlighting changes over the past 3–5 years, is summarized below.

### 6.1. First-Line Treatment for Unresectable/Metastatic Melanoma

The NCCN (US) and ESMO (European) guidelines recommend checkpoint inhibitors as the first-line treatment for most patients. According to the NCCN guidelines, combination checkpoint immunotherapy is generally preferred over PD-1 monotherapy in eligible patients. This is due to improvements in response rate, progression-free survival, and overall survival [60]. Nivolumab plus ipilimumab is a Category 1 recommended regimen, and nivolumab plus relatlimab is also designated as a Category 1 recommended option (regardless of BRAF-mutation status) in the latest NCCN update. Anti-PD-1 monotherapy (nivolumab or pembrolizumab) also maintains a Category 1 recommendation and is particularly suitable for patients who may be unable to tolerate the toxicity of combination therapy. For BRAF-mutation-positive melanomas, BRAF/MEK inhibitor therapy is included as a Category 1 recommended first-line treatment. The guidelines state that initiating BRAF/MEK-targeted therapy may achieve rapid disease control in cases of high tumor burden or rapidly progressing symptomatic disease. However, when the disease is not acutely life-threatening, many experts recommend selecting immunotherapy first, even for patients with BRAF mutations, considering the potential for long-term remission (a discussion on sequencing follows).

### 6.2. Adjuvant Therapy (Resected Stage III/IV)

These guidelines universally recommend adjuvant systemic therapy for resected stage IIIB–IIID and IV melanomas. Options include anti-PD-1 immunotherapy (nivolumab or pembrolizumab administered for 1 year) for all patients, and dabrafenib plus trametinib administered for 1 year as an alternative therapy for patients with BRAF mutation. These recommendations are based on clinical trials that have demonstrated improvements in recurrence-free survival. The NCCN and ESMO list immunotherapy and targeted therapy as Category 1 adjuvant therapies for BRAF-mutated melanoma. Patient factors and preferences (e.g., tolerability, route of administration, and desire to avoid fever risk with BRAF/MEK inhibitors versus immune-related side effects) guide the choice. Recent updates include the addition of nivolumab and pembrolizumab as adjuvant therapies for specific high-risk stage IIB/IIC patients [61].

### 6.3. Neoadjuvant Therapy

Although not yet a standard treatment per the guidelines, neoadjuvant therapy is gaining attention. The NCCN guidelines report that preclinical trials of preoperative anti-PD-1 therapy or ipilimumab plus nivolumab demonstrated a correlation between high pathological response rates and improved prognosis [60]. Based on these results, current guidelines recommend participation in preclinical trials for patients with resectable stage III melanoma. Ongoing studies demonstrating event-free survival benefits may lead to formal guideline recommendations in the near future.

### 6.4. Central Nervous System Metastases

Owing to the frequent occurrence of brain metastases in malignant melanoma, the guidelines provide specific recommendations. For asymptomatic patients with brain metastases, systemic therapy with PD-1 ± CTLA-4 inhibitors is recommended as the first-line option. This recommendation is based on data showing that combination immunotherapy can achieve a brain response rate of approximately 50–55%. Targeted therapy has also demonstrated high intracranial activity in patients with BRAF mutations (approximately a 58% response rate in brain metastases in the COMBI-MB trial). However, the duration of response may be relatively short. Therefore, surgery and stereotactic radiosurgery are often performed in addition to drug therapy. Recent guideline revisions have emphasized the role of immunotherapy, even for brain metastases. The CheckMate-204 and ABC trials demonstrated sustained intracranial responses to nivolumab and ipilimumab.

### 6.5. Special Populations

The guidelines also address subtypes, such as uveal melanoma (tebentafusp is recommended for patients who are HLA-A*02:01-positive; checkpoint inhibitor efficacy is limited) and mucosal melanoma (immunotherapy remains recommended, but response rates are slightly lower than those in cutaneous melanoma; KIT mutation testing is recommended). Guidance is also included for treating patients with autoimmune diseases or those who have undergone organ transplantation, requiring individualized treatment, frequent consultation, and cautious use of ICIs.

### 6.6. Post-Progression Management

These guidelines have been updated to reflect new options after disease progression. For example, for patients who have progressed on anti-PD-1 therapy, the NCCN lists ipilimumab or the previously unused combination of nivolumab plus ipilimumab as a treatment option. If approved, TIL therapy should be included in the guidelines as a third-line treatment. For advanced melanoma, participation in clinical trials is recommended at each line of treatment.

## 7. Biomarkers for Treatment Selection and Response Monitoring

Identifying biomarkers is critical for optimizing melanoma treatment by selecting the appropriate therapy for the right patient, and for monitoring treatment response and recurrence. To improve clinical applicability, melanoma biomarkers can be prioritized based on their level of implementation as follows: (i) clinically actionable, where they directly alter treatment availability or selection; (ii) prognostic, where they are used for risk stratification and counseling; and (iii) exploratory or research, where they are informative but not routinely required for decision-making.

Examples of clinically actionable biomarkers include BRAF V600 mutation status, which is essential for determining eligibility for BRAF/MEK-targeted therapy at diagnosis of advanced disease. In certain situations, subtype- or context-specific actionable factors, such as HLA-A*02:01 for tebentafusp in uveal melanoma and KIT alteration testing in mucosal/acral melanoma, may influence access to specific therapies or trials.

Prognostic biomarkers (e.g., LDH and, in some contexts, S100 and CRP) inform baseline risk and expected outcomes, but they do not mandate a specific regimen by themselves.

Exploratory/research biomarkers (e.g., PD-L1, TMB, gene expression profiles/immune signatures, ctDNA for minimal residual disease [MRD] and early relapse detection, and toxicity predictors for irAEs) are increasingly used for trial stratification and hypothesis generation, whereas their routine use remains limited or evolving. The key biomarkers and their current utility are as follows:

### 7.1. BRAF-Mutation Status

Testing for the BRAF V600 mutation at diagnosis is essential for advanced melanoma. This identifies patients eligible for BRAF/MEK-targeted therapy and guides treatment decisions [62]. BRAF-mutation-positive (V600E/K) status enables targeted therapy options, whereas patients with BRAF wild-type tumors receive immunotherapy as a systemic treatment. BRAF status generally does not predict the benefit of immunotherapy (BRAF-mutant and wild-type melanomas respond similarly to PD-1/CTLA-4 inhibitors); however, it is a prognostic factor and clearly predicts responses to BRAF/MEK inhibitors.

### 7.2. PD-L1 Expression

Tumor PD-L1 expression has limited utility in melanoma compared with other cancer types [63]. Since PD-1 therapy is effective in melanoma regardless of PD-L1 status, neither NCCN nor ESMO mandates PD-L1 testing for patient selection. Therefore, PD-L1 expression can serve as a prognostic marker, as patients with PD-L1-positive tumors often exhibit a more inflammatory TME at baseline [64]. Clinical studies frequently report PD-L1 status, which correlates with response rates; however, its use in clinical practice is optional.

### 7.3. Tumor Mutation Burden (TMB)

Melanoma typically exhibits one of the highest TMBs among cancer types, largely owing to UV-induced DNA damage [65]. High TMB correlates with the potential for response to immunotherapy. Additional mutations generate a greater number of targetable neoantigens. Indeed, TMB is a key factor underlying the approval of pembrolizumab for high-TMB tumors, regardless of histology [66]. Approximately all melanoma tumors, particularly cutaneous melanoma, exhibit high TMB levels; therefore, routine TMB testing is generally unnecessary. However, melanomas with an extremely low TMB (more common in the acral lentiginous and mucosal subtypes) may show lower response rates to PD-1 inhibitors. Because most cutaneous cases fall into the high-TMB category, TMB remains a primary research biomarker.

### 7.4. Gene Expression Profile (GEP) and Immune Signatures

Recent studies have identified gene expression signatures that can predict immunotherapy outcomes. For example, high expression of the IFN-γ gene signature within tumors—an “inflammatory” gene profile—suggests pre-existing T-cell inflammation and correlates with a favorable response to checkpoint inhibitors, whereas a “cold” tumor signature predicts resistance [67]. Some assays, such as NanoString’s Tumor Inflammation Signature and other GEP tests, are under evaluation but have not yet met clinical standards. However, they are useful for patient stratification in clinical trials and may become valuable if treatments based on immune phenotypes are established in the future.

### 7.5. Serum Biomarkers (e.g., LDH)

LDH is an established prognostic biomarker for melanoma. Elevated serum LDH levels correlate with increased tumor burden and poor prognosis and are incorporated into the definition of M1c/M1d staging [68]. Although LDH does not directly dictate treatment selection, it is useful for risk stratification; patients with normal LDH levels show favorable outcomes across all treatments, whereas those with elevated LDH levels may require more aggressive combination therapy. Other blood-based markers, such as S100 protein and CRP, are also prognostic factors [69]. Recent data suggest that baseline ctDNA levels correlate with tumor burden and prognosis [70].

### 7.6. ctDNA

ctDNA is a promising tool for monitoring treatment response. In melanoma, studies have shown that patients who achieve undetectable ctDNA levels early in treatment demonstrate significantly better outcomes [71]. Conversely, rising ctDNA levels may indicate recurrence or progression earlier than that observed in clinical imaging. For example, in adjuvant therapy, postoperative ctDNA detection predicts a high risk of recurrence, whereas ctDNA disappearance during treatment is correlated with treatment efficacy [72]. Although not yet routine, ctDNA testing holds potential for guiding follow-up, early detection of recurrence, and timing of treatment resumption, advancing toward clinical application.

### 7.7. Toxicity Biomarkers

Research on markers predicting irAEs from checkpoint therapy (e.g., specific HLA subtypes, T-cell repertoire characteristics, and cytokine changes) is ongoing; however, no clinical application has yet been established.

### 7.8. Other Exploratory Biomarkers

MHC class I/II expression in tumor cells is essential for T-cell recognition. Loss of HLA expression has been observed in some resistant melanomas, potentially explaining rare immunotherapy failures and suggesting the utility of assessing HLA expression in tumors [73]. Furthermore, the gut microbiota is associated with responsiveness to immunotherapy. Recent studies have indicated that patients with specific gut bacterial compositions exhibit enhanced efficacy of PD-1 inhibitors [74]. This is an intriguing area with potential implications for enhancing immunotherapy efficacy through probiotics or dietary interventions.

## 8. Real-World Challenges and Future Directions

Despite the remarkable advances in melanoma pharmacotherapy, numerous practical challenges remain. These include managing treatment resistance, addressing treatment toxicity, determining the optimal treatment sequence, and ensuring that patients in clinical settings receive the full benefits demonstrated in trials. This section discusses these challenges and outlines directions for further improvements in patient outcomes.

### 8.1. Treatment Resistance

#### 8.1.1. Resistance to Targeted Therapies

Almost all patients treated with BRAF/MEK inhibitors eventually experience tumor progression owing to acquired resistance mechanisms (as described above) [20]. Clinically, when resistance develops, switching to immunotherapy that was not used during the initial treatment is typically the next option. Future strategies to address resistance may include next-generation targeted agents (e.g., ERK inhibitors or inhibitors of other pathway components) or combination therapies at the time of disease progression. One approach gaining attention in clinical trials is the re-administration of BRAF/MEK inhibitors after a treatment interruption period (“drug holiday”). Case reports have indicated tumor resensitization after treatment interruption, suggesting the potential utility of treatment cycles [75]. Furthermore, in patients with oligoprogression (progression at a single site) receiving targeted therapy, local treatment (surgery or radiotherapy), combined with continued targeted agent administration, may control disease at other sites.

#### 8.1.2. Immunotherapy Resistance

A significant proportion of patients (approximately 40–60%) do not respond to first-line PD-1-based immunotherapy (primary resistance). Furthermore, among the responders, some relapse occurs after an initial response (acquired resistance). Mechanisms of immunotherapy resistance are diverse. Loss of antigen presentation function (e.g., β2-microglobulin or HLA gene mutations) can prevent tumor cells from being recognized by T-cells. An immunosuppressive TME, characterized by numerous Treg cells, MDSCs, and inhibitory cytokines, inhibits effective immune responses. On the other hand, overexpression of alternative checkpoint molecules on T-cells (e.g., TIM-3, TIGIT) may mediate resistance [76]. Identifying these mechanisms in individual patients remains challenging. However, research is underway to tailor subsequent treatments. For example, in patients who progress to PD-1 inhibitor treatment, strategies include adding CTLA-4 inhibitors. Ipilimumab activates different T-cell populations or expands immune responses, yielding additional responses in some cases [77]. Clinical trials are also investigating combination therapies using PD-1 inhibitors with novel agents, such as anti-TIGIT antibodies or IL-2 variants, to overcome resistance. Treatment sequencing is critical and is discussed later in this paper. A molecular-level understanding of resistance, achieved through repeated biopsies of progressive tumors and genomic and transcriptomic analyses, is key to developing next-generation therapies.

#### 8.1.3. Adjuvant Therapy and Secondary Resistance

Adjuvant treatment strategies for recurrence after PD-1 therapy have been actively debated. Options include re-administration of ipilimumab or participation in clinical trials, such as studies evaluating the efficacy of PD-1 re-administration after a prolonged interval. Similarly, resistance patterns in brain metastases, which have distinct blood–brain barriers and microenvironments, are being studied separately. Combining systemic therapy with radiation therapy, which may have immunomodulatory effects, represents a strategy to overcome resistance in the brain [78]. Future directions for resistance management include rational combination therapies (e.g., adding epigenetic modulators to “heat up” “cold” tumors), personalized treatment based on resistance profiling, and adaptive trial designs that modify treatment in real time based on early biomarkers, such as rising ctDNA levels or gene expression changes indicating resistance.

### 8.2. Toxicity Management

Melanoma therapies can cause significant toxicity and require active management, particularly in an adjuvant setting, where curative intent is assumed, and higher toxicity may be tolerated. Key challenges and management strategies are discussed below.

#### 8.2.1. irAEs

Checkpoint inhibitors activate the immune system against cancer and against healthy tissues, causing irAEs. These toxicities can affect any organ but are particularly common in the skin (rash), colon (colitis), endocrine glands (hypothyroidism, hypophysitis), liver (hepatitis), and lungs (pneumonitis) [79]. Combination immunotherapy has a higher incidence of grade 3–4 irAEs (up to approximately 55% in studies such as CheckMate-067), whereas PD-1 monotherapy causes grade 3–4 irAEs in approximately 10–20% of cases [9]. Management of irAEs requires multidisciplinary care; immunosuppressive steroids are the first-line treatment for moderate to severe irAEs, and if improvement is not observed, additional immunosuppressants (e.g., infliximab for colitis and mycophenolate for hepatitis) are used [80]. Guidelines (ASCO, ESMO, and SITC) provide severity classifications and treatment algorithms for each type of toxicity. Patient education is essential for early reporting of symptoms (e.g., diarrhea in patients undergoing immunotherapy should prompt evaluation of colitis). In real-world clinical practice, a key challenge is ensuring that community oncologists and patients recognize these side effects and promptly address them to avoid serious complications. Another challenge is permanent endocrine dysfunction (e.g., adrenal insufficiency and type 1 diabetes), which requires lifelong hormone replacement therapy. Endocrinological follow-up is essential for patient survival. Advances in research on predicting irAEs, such as baseline autoantibodies or T-cell profiles, may eventually enable personalized risk stratification.

#### 8.2.2. Targeted Therapy Side Effects

BRAF/MEK inhibitor combination therapy has a distinct toxicity profile, including fever syndrome (occurring in approximately 50% of patients receiving dabrafenib/trametinib), photosensitivity rash (particularly with vemurafenib), arthralgia, hepatotoxicity, and, less frequently, serious adverse effects such as cardiomyopathy and ocular toxicity (retinal detachment and MEK inhibitor-associated central serous chorioretinopathy) [81,82]. Effective management requires patient counseling (e.g., strict sun protection to prevent photosensitivity reactions) and prompt intervention for fever, which may necessitate dose interruption or short-term administration of steroids or nonsteroidal anti-inflammatory drugs to break the fever cycle. Experienced oncologists have developed strategies for dose reduction and treatment interruption to manage these side effects while maintaining efficacy. Supportive care, such as aggressive hydration and antipyretic administration, is standard. As the side effects of targeted therapy are generally reversible, unlike some irreversible immunotherapy effects, oncologists often adopt intermittent dosing approaches to improve tolerability [83]. Practical considerations include compliance with and adherence to targeted oral therapy. Patients must be educated to continue treatment as prescribed, even when feeling well, and to consult their healthcare team before discontinuing medication owing to side effects.

#### 8.2.3. Quality of Life and Long-Term Toxicity

As patient survival increases, the importance of chronic treatment-related toxicity also increases. For example, immunotherapies may cause persistent arthritis or neuropathy, requiring co-management with rheumatologists or neurologists [84]. Interest in dose-reduction strategies is also growing. Whether treatment can be discontinued to reduce cumulative toxicity in patients achieving long-term complete remission remains an important clinical question. Some trials (e.g., SAFE-STOP) have explored discontinuation of PD-1 therapy 1–2 years after a response in responders [85]. Initial data have shown that many patients maintain remission after treatment discontinuation, potentially avoiding side effects and costs. Current guidelines acknowledge that continuing immunotherapy beyond 2 years is not necessarily required for patients with a complete response, although this decision should be individualized.

### 8.3. Treatment Sequencing and Integration

One practical dilemma in melanoma treatment is the optimal sequencing of available therapies. In patients with BRAF mutations, immunotherapy and targeted therapy are viable treatment options. Recent evidence clarifies this issue; however, actual decision-making requires consideration of disease progression rate and patient status.

#### 8.3.1. Immunotherapy First vs. Targeted Therapy First

Two randomized trials examined the treatment sequencing of BRAF-mutated metastatic melanoma. The US DREAMSeq (EA6134) and European SECOMBIT trials compared a group starting with immunotherapy (nivolumab plus ipilimumab in DREAMSeq) and switching to a BRAF/MEK inhibitor therapy upon progression with a group using the reverse sequence [86]. These trials demonstrated a clear benefit for the group that initiated immunotherapy. In the DREAMSeq trial, the 2-year survival rate (approximately 72%) in the group initially receiving nivolumab plus ipilimumab exceeded that in the group starting with dabrafenib plus trametinib (approximately 52%) [87]. Similarly, the SECOMBIT trial reported improved 4-year survival rates with immunotherapy as the initial treatment, confirming that immunotherapy as a first-line therapy yields more sustained long-term benefits. The biological rationale is that patients who first receive targeted therapy may miss the opportunity for immunotherapy if their disease progresses rapidly and causes organ dysfunction. Conversely, initiating with immunotherapy allows for sustained control, with targeted therapy serving as a “rescue” option for patients who do not respond to immunotherapy. The current standard of care, as reflected in the NCCN guidelines, favors immunotherapy as the first-line therapy for most BRAF-mutated cases unless urgent tumor shrinkage is required.

#### 8.3.2. When Targeted Therapy Should Be Used First

Some scenarios exist in which the initiation of targeted therapy is reasonable. For example, in patients with a high tumor burden presenting with visceral crisis (such as large lesions causing organ dysfunction), immediate tumor shrinkage with BRAF/MEK inhibitors may be beneficial. BRAF/MEK inhibitors often produce a rapid response within weeks, whereas immunotherapy may take longer to show an effect and may sometimes cause pseudo-progression or worsen inflammation. An approach currently under investigation (arm 3 of the SECOMBIT trial) involves short-term BRAF/MEK inhibitor treatment (8 weeks) for tumor reduction, followed by a switch to immunotherapy before resistance develops [86]. This “induction therapy” strategy aims to achieve the dual benefits of rapid tumor control and sustained immunotherapy efficacy. The initial results suggest feasibility, but implementation outside clinical trials is complex.

#### 8.3.3. Treatment Sequence After First-Line Therapy

If the disease progresses after initial immunotherapy, subsequent systemic therapy options include targeted therapy (for patients with BRAF mutations) or ipilimumab-based combination therapy (for patients starting PD-1 monotherapy). Conversely, if the disease progresses with targeted therapy (and immunotherapy has not been administered), immunotherapy is typically used next and may still be effective. Notably, some patients who fail BRAF/MEK therapy have demonstrated favorable responses to subsequent PD-1 ± CTLA-4 therapy. One challenge is that long-term targeted therapy may alter the TME (e.g., by inducing T-cell exclusion). However, evidence also suggests that “withdrawal” from targeted drugs may allow tumors to regain sensitivity to immunotherapy over time [88]. Alternatively, disease progression may occur after immunotherapy and targeted therapy. Options include clinical trials (e.g., new agents or TIL therapy) or rechallenge with previously used drug regimens if sufficient time has elapsed.

#### 8.3.4. Integration of Local Therapies

The roles of surgery and radiation therapy in treatment sequencing should not be overlooked. Oligometastatic melanoma can be managed with metastasectomy or stereotactic radiotherapy combined with systemic drug therapy. For example, in a patient with a single residual metastasis during immunotherapy, resection of the lesion may be considered potentially curative. Radiotherapy has synergistic effects with immunotherapy, and abscopal effects have been reported in melanoma [78].

#### 8.3.5. Transition from Adjuvant to Metastatic Setting

Another sequencing issue concerns patients who relapse after adjuvant therapy. For patients who relapse after receiving pembrolizumab as an adjuvant therapy, the question arises regarding whether to repeat immunotherapy or transition to targeted therapy if BRAF-mutated melanoma is detected. Current standard practice often involves initiating combination therapy with ipilimumab for tumors that progress on PD-1 inhibitors, or selecting targeted therapy if applicable. However, the optimal approach remains unclear and is currently under investigation. Similarly, for patients who relapsed after receiving dabrafenib/trametinib as an adjuvant therapy, immunotherapy is typically used as the next step. This is because many patients respond to PD-1 inhibitors even if targeted therapy fails during the adjuvant phase.

In summary, treatment sequencing involves subtle nuances and varies among patients. A general trend is to introduce immunotherapy early to ensure durable disease control, strategically use targeted therapy for rapid reduction or as a secondary treatment in BRAF-mutated disease, and incorporate local therapy when beneficial. Multidisciplinary tumor boards are useful in determining the optimal sequence for each patient. Ongoing clinical trials are exploring adaptive treatment sequencing, in which therapies are switched based on early markers of response or resistance, to further individualize the treatment process.

## 9. Future Outlook

The outlook for melanoma treatment continues to evolve rapidly. Future prospects include biomarker-guided personalized treatment and prevention of disease recurrence. Accordingly, new approaches are expected to be developed in the near future. For example, if TIL therapy is approved for advanced melanoma, it could become a lifeline for patients who have exhausted existing treatment options.

An expansion of combination strategies is anticipated. For instance, ongoing Phase III trials are being conducted to evaluate combinations of PD-1 and TIGIT inhibitors, whereas other studies are aimed at investigating the addition of novel agents such as HDAC inhibitors to immunotherapy. Should any of these demonstrate clear efficacy, the standard treatment for specific patients could shift toward triple combination therapy.

Biomarker-guided treatment is a primary goal. Within the next 5 years, genomic and immunological biomarker profiles may enable identification of patients who should receive combination immunotherapy versus monotherapy, as well as those who may benefit from the addition of emerging agents. The integration of artificial intelligence and machine learning to predict responses from pathological images and genomic data is under investigation in melanoma and may aid in clinical decision-making.

Another frontier involves the prevention of melanoma recurrence in high-risk patients. Research on personalized neoantigen vaccines suggests a shift from uniform approaches toward individualized adjuvant therapies. Patients with specific tumor signatures may receive a vaccine plus PD-1, whereas others may require no additional therapy.

Finally, management of melanoma as a chronic, controllable disease is becoming a reality for some patients. Given that approximately half of patients with advanced melanoma receiving combination immunotherapy may become long-term survivors, refinement of survivor support programs and long-term monitoring strategies are essential. The aforementioned dose-reduction studies and chronic toxicity management research will be key to preserving patients’ quality of life.

In summary, current drug therapies have dramatically improved the prognosis of melanoma. However, ongoing research, clinical trials, and real-world data will continue to guide best practice. The ultimate goal is to increase the proportion of patients achieving sustained remission or cure, minimize treatment-related complications, and deliver personalized therapy tailored to tumor and patient characteristics.

## 10. Limitations

This article is a narrative review by a single author, not a systematic review. Although the goal was to provide a balanced synthesis based on representative and recent evidence, the selection of studies may be influenced by publication bias and evolving guideline updates. Therefore, conclusions, particularly those regarding emerging therapies, should be interpreted in the context of ongoing trials and future evidence.

## 11. Conclusions

Melanoma was once considered an “incurable cancer,” but today, a wide range of effective systemic therapies exists. ICIs (anti-PD-1, anti-CTLA-4, and newer LAG-3 inhibitors) and molecularly targeted agents (BRAF and MEK inhibitors) form the current treatment backbone and prolong survival in many patients. Furthermore, novel therapies, ranging from personalized vaccines to cell therapies and oncolytic viruses, are emerging and offer the potential for further improvement of treatment outcomes, particularly for patients who are unresponsive to existing therapies. Clinical guidelines are rapidly incorporating these advances; however, their practical application requires careful consideration of patient and disease factors (e.g., mutational status, disease burden, and comorbidities).

Oncology and dermatology specialists must stay abreast of evolving evidence, including subtle differences in treatment sequencing and combination therapies, to maximize patient benefit. Research continues to address key challenges associated with drug resistance and toxicity. Treatment options for melanoma will likely expand further in the coming years, as numerous clinical trial results continue to be published and novel therapies (such as autologous T-cell immunotherapy and next-generation immunomodulators) continue to be tested. Through continuous innovation and careful clinical application, most patients with melanoma are anticipated to reach a stage at which sustained remission or cure is achievable, marking one of the most significant turning points in the history of cancer.

## Figures and Tables

**Table 1:** Novel therapies currently under investigation for melanoma, listed by category: molecularly targeted drugs, immune checkpoint molecules, and other targets.

Classification	Target
Molecular Targeted Therapy	NRAS (Currently Substituted with MEK Inhibitors)
	c-KIT
	NTRK
	VEGF
Immune Checkpoint Inhibitors	LAG3
	TIGIT
	TIM-3
Others	Cytokine Therapy (IL-2, IL-21, IL-15)
	Personalized Cancer Vaccines (mRNA Vaccines, Peptide Vaccines, DNA Vaccines)
	Adoptive Cell Therapy (Tumor-infiltrating lymphocytes (TILs), T-cell receptors (TCRs))
	Bispecific Antibodies and T-Cell Engagers (gp100/CD3)
	Oncolytic Viruses (T-VEC)
	PRP Inhibitors
	Metabolic and Epigenetic Targets (IDO Inhibitors, HDAC Inhibitors)
	PAI-1 Inhibitors

## Data Availability

This article is authored by a single author, and all cited literature is listed in the references.

## Abbreviations

ICI	Immune checkpoint inhibitors
mRNA	Messenger ribonucleic acid
TCR	T-cell receptors
T-VEC	Talimogene laherparepvec
